# Early detection of fungal infection of Arabidopsis and brassica by Raman spectroscopy

**DOI:** 10.3389/fpls.2025.1649206

**Published:** 2025-08-15

**Authors:** Song-Yi Kuo, Ling-Ying Chiu, Ekta Jain, Gajendra Pratap Singh, Muhammad Nabil Syafiq Bin Jamaludin, Rajeev J. Ram, Nam-Hai Chua

**Affiliations:** ^1^ Temasek Life Sciences Laboratory, 1 Research Link, National University of Singapore, Singapore, Singapore; ^2^ Disruptive and Sustainable Technologies for Agricultural Precision, Singapore-MIT Alliance for Research and Technology, Singapore, Singapore; ^3^ Research Laboratory of Electronics, Massachusetts Institute of Technology, Cambridge, MA, United States

**Keywords:** *Arabidopsis thaliana*, Brassica vegetables, biotic stress, fungal infection, pattern-triggered immunity (PTI), Raman spectroscopy, early diagnosis

## Abstract

Here, we used Raman spectroscopy to characterize the effects of chitin treatment and fungal inoculations on *Arabidopsis thaliana* and Brassica vegetables. Chitin, a recognized fungal pathogen-associated molecular pattern (PAMP), elicited a dose dependent positive Elicitor Response Index (ERI) in wild-type Arabidopsis. Mutant plants lacking chitin receptors (*cerk1* and *lyk4/5*) displayed minimal ERI, whereas *fls2* mutant deficient in the bacterial-specific flg22 receptor was hyper-responsive. These results confirm critical role of chitin receptors in activating downstream pathways and highlighting distinct responses in two separate pattern-triggered immunity (PTI) systems. Inoculations of *Colletotrichum higginsianum* and *Alternaria brassicicola* induced significant changes in Infection Response Index (IRI) values, with the former giving positive IRI at 12–48 hours post-inoculation whereas the latter exhibited a transient negative IRI before transitioning to positive values. Notably, Raman shifts could predict fungal infection before the appearance of visible symptoms, establishing Raman shifts as a potential early diagnostic marker. Comparative analyses of infected Brassica vegetables revealed varied sensitivity to fungal pathogens and a correlation between symptom severity and IRI values. Furthermore, randomized controlled trials validated the reliability of Raman technology for early, pre-symptomatic detection of fungal infections, achieving an accuracy rate of 76.2% in Arabidopsis and 72.5% in Pak-Choy (*Brassica rapa chinensis*). Principal component analysis differentiated Raman spectral features associated with fungal and bacterial infections, emphasizing their unique profiles and reinforcing the utility of Raman spectroscopy for early detection of pathogen-related plant stress. Our work supports the application of non-invasive diagnostic techniques in agricultural practices, enabling timely intervention against crop diseases.

## 1 Introduction

Food security is challenged by increasing geopolitical tensions and rapid climate change. About 50% and 25-40% of losses in crop yield world-wide are attributed to abiotic and biotic stressors, respectively (Kumar et al., 2022). Early and rapid diagnosis of the responsible stressor may allow timely intervention and implementation of remedial measures in the field. By enabling timely mitigation, guiding resource deployment, and informing predictive models, it minimizes crop losses and enhances resilience against future challenges (Ristaino et al., 2021).

Incorporating advanced technology into agricultural practices is essential for safeguarding food security. Traditional plant disease detection methods, including visual scouting and laboratory analysis, provide essential confirmation of disease presence and pathogen identification. Serological assays, like ELISA, offer high-throughput capabilities, while qPCR confirmation analysis provides sensitive quantification of pathogens. However, these methods rely on visible symptoms or require sample processing, delaying detection until later stages of disease propagation. Innovative methods, such as volatile compound analysis and remote sensing, offer non-invasive characterization of plant health and can detect subtle changes before symptoms appear, allowing for earlier intervention. This timing is crucial in managing polycyclic diseases and preventing widespread crop loss. Furthermore, techniques such as bio-photonics and advanced imaging provide real-time, *in situ* analysis, minimizing disturbance to the plant and offering more comprehensive spatial-temporal data, complementing the accuracy of traditional methods with improved speed and preventative capabilities (Martinelli et al., 2015).

Recent research has underscored the effectiveness of Raman spectroscopy (RS) in early diagnosis of both biotic and abiotic stresses in crops. This non-invasive laser technique enables rapid analysis without requiring chemical labelling or compromising the integrity of the sample, making it a cost-effective solution for breeders (Saletnik et al., 2024).

RS analyzes light scattering from molecules. When a photon interacts with a molecule, it can scatter elastically (no energy change) or inelastically (Raman scattering, with an energy shift). This energy shift, or Raman shift is specific to the molecule’s vibrational states (Langer et al., 2019). The resulting Raman spectrum, a plot of intensity versus Raman shift, provides a unique fingerprint for each molecule and its functional groups whereas peak heights within the spectrum correlate with the concentrations of specific molecules in the sample. The integration of qualitative and quantitative data from Raman spectrum allows profiling the vibrational states of biological molecules (Saletnik et al., 2021). The ability to acquire spectra representing detailed molecular information from intact tissues, with minimal interference from vibrational bands of water, renders this technology a powerful analytical tool for studying biological samples (Gierlinger and Schwanninger, 2007; Orkoula and Kontoyannis, 2014).

RS has emerged as a useful tool for diagnosing plant diseases by identifying pathogen-induced changes in plant metabolite profiles. This technology has been applied effectively to detect fungal infections in important crops like sorghum, wheat, and corn. RS coupled with chemometrics has been used to identify the infection of four fungal pathogens on maize kernels or fungal infected sorghum grains. Surface-enhanced Raman spectroscopy (SERS) has enabled the detection of Fusarium wilt of banana (FWB) by fingerprinting, an assay based on the database that includes the spectral characteristics of the pathogen. In combination with multivariate statistical analysis, RS has also been used for identifying a bacterial disease, Huanglongbing, in citrus trees. *Candidatus Liberibacter solanacearum* (Lso) infection on tomato can also be detected 3 weeks following infection. The distinct spectral features produced by different viruses enable RS to differentiate infections caused by various viral combinations or individual viruses on rose, wheat or tomatoes. The method can also identify grapevine infections attributable to grapevine fanleaf virus (GFLV) and grapevine stem pitting-associated virus (GRSPaV) with 100% and 80% accuracy, respectively, before appearance of phenotypic symptoms (Weng et al., 2021).

In most of these studies Raman spectra can detect metabolic changes in plants during pathogen interactions. Metabolites with resonance structures contribute significantly to the spectral features observed. For instance, bands associated with carotenoids appear in the ranges of 1001–1151 cm^-1^ and 1520–1550 cm^-1^. The resonance structures of these metabolites play crucial roles in their biological functions including light absorption in photosynthetic systems and antioxidant activity under stress conditions (Chung et al., 2021).

To sense biotic stresses from the environment, plants have developed a system that recognizes pathogen-associated molecular patterns (PAMPs), which are composed of conserved immunogenic structures derived from pathogens, including bacteria, fungi, and viruses. PAMPs can be detected by Pattern recognition receptors (PRRs) locate on cell-surface. These receptors are receptor-like proteins (RLPs) or receptor-like kinases (RLKs) which transmit information about the extracellular stimuli to the cytosol to render a series of corresponding PTI as the first layer of plant defense system (Van Der Burgh and Joosten, 2019).

Flagellin Sensing 2 (FLS2) and the elongation factor Tu Receptor (EF-Tu receptor, EFR) are two well studied RLKs in *Arabidopsis thaliana*. FLS2 specifically detects the flagellin-derived immunogenic peptide flg22, whereas EFR recognizes elf18, a peptide from the bacterial EF-Tu (Gómez-Gómez and Boller, 2000; Zipfel et al., 2006). Binding of flg22 strengthens the dimerization between FLS2 and another RLK, Brassinosteroid insensitive 1-associated receptor kinase (BAK1) resulting in mutual phosphorylation thus triggering the cytosolic immune response (Chinchilla et al., 2007). Besides the similar heterodimerization, EFR has also been proposed to activate BAK1 via an allosteric regulation (Mühlenbeck et al., 2024).

In contrast to bacteria-derived immunogenic peptides, defence response against fungal infection is triggered by chitin, a key component of fungal cell walls, which serves as a PAMP for fungi. Chitin elicitor receptor kinase 1 (CERK1, LysM receptor-like kinase 1, LYK1) is the first identified chitin receptor that physically interacts with chitin to initiate the MAPK signaling cascade. Mutations in *CERK1* greatly reduce ROS production which is part of plant immunity response against fungi (Miya et al., 2007). Two other LysM receptor-like kinases, LYK4 and LYK5 have been identified as the core components of the chitin receptor complex. Besides their redundant role in chitin-responsive MAPK activation, LYK5 could also induce phosphorylation of CERK1 and LYK4 and is indispensable for ROS production in response to chitin. Mutations in either *LYK4* or *LYK5* renders plants partially insensitive to chitin treatment whereas a *lyk4/5* double mutant shows little or no chitin response (Cao et al., 2014; Wan et al., 2008).

Upon recognizing PAMPs, PRRs activate various downstream signaling pathways including the Mitogen-Activated Protein Kinase (MAPK) pathways. These pathways, which are highly conserved across eukaryotes, serve as essential signaling networks that regulate a wide range of physiological processes in plants and they effectively transducing external stimuli into appropriate cellular responses. Particularly important for mediating responses to both biotic and abiotic stresses, MAPK pathways consist of 3 sequentially activated protein kinases: MAPK, MAPK kinase (MAPKK), and MAPKK kinase (MAPKKK). This tiered structure facilitates signal amplification and specificity (Sun and Zhang, 2022).

The activation of MAPK pathways by PAMPs and other stress signals induces several downstream effects, including the production of reactive oxygen species (ROS), activation of defence-related genes, and the establishment of systemic acquired resistance (SAR) (Genot et al., 2017; Mersmann et al., 2010). In response to increased ROS levels, plants may enhance synthesis of protective pigments. For instance, carotenoids and flavonoids, known for their antioxidant properties, often increase in abundance to scavenge ROS and mitigate oxidative damage, helping to stabilize the photosynthetic apparatus. Similarly, flavonoids such as anthocyanins can be upregulated in response to various stresses, providing photoprotection by absorbing excess light and shielding tissues from injury. Conversely, chlorophyll content may decline under stressful conditions leading to chlorosis and consequently reduced photosynthetic efficiency (Han et al., 2012; Xu and Rothstein, 2018).

As fungal infestations pose significant challenges to many agricultural systems, implementing effective early detection strategies is crucial to prevent crop loss. Timely intervention facilitated by early detection technology can help minimize chemical use, enhance food security, and promote economic sustainability in farming practices. In previous studies, we have demonstrated the potential of RS as an early diagnosis system based on detecting metabolites changes mediated by innate immunity against bacteria (Chung et al., 2021). Here, we extend and broaden the application of this early diagnosis system to detect fungal infections in plants.

## 2 Materials and methods

### 2.1 Plant preparation

*Arabidopsis thaliana* wild type (Col-0) and receptor mutants, *lyk4/5* (Cao et al., 2014)*, cerk1* (GABI_096F09) and *fls2* (salk_062054) were grown at 22°C with 60% relative humidity (RH) with a short-day photoperiod of 10 hours light and 14 hours darkness under a light intensity of 100 µmol/m²/s. Three-week-old plants were used for fungal inoculations whereas four- to five-week-old plants were used for chitin treatments. Plants of Pak-Choy (*Brassica rapa chinensis*) and Choy-Sum (*Brassica rapa* var. *parachinensi*s) were grown at 22°C with 60% RH with a long-day photoperiod of 16 hours light and 8 hours darkness under a light intensity of 125 µmol/m²/s. Sixteen- to eighteen-day-old plants were used for fungal inoculations.

### 2.2 Preparation and infiltration of chitin emulsion

A water solution containing 100 mM chitin (Merck, Cat. No. C9752-1G) was emulsified by sonication for 2 hours on ice. The resulting chitin emulsion was diluted with distilled water to the desired concentrations before being infiltrated into leaves using a needleless syringe as previously described (Chung et al., 2021). Distilled water without chitin was used for mock treatment.

### 2.3 Fungal inoculum and inoculation

*Colletotrichum higginsianum* and *Alternaria brassicicola* were grown on Potato Dextrose Agar (PDA, Sigma-Aldrich, Cat. No. P6685) until spores were generated. Spores were suspended in distilled water containing 0.02% Silwet L-77 and spore concentration was determined using a hemocytometer. A water solution containing the specified spores per milliliter (spores/mL) was prepared. A drop (approximately 10 µl) of the fungal spore solution was deposited on both sides of the leaf midrib whereas a solution with the corresponding concentration of Silwet L-77 was applied for mock inoculations. Inoculated plants were then covered with a transparent lid to maintain high RH.

### 2.4 Sample collection for analysis by Raman spectroscopy

We used a custom-built Raman spectroscopy system as previously described (Chung et al., 2021) with slight modifications. The Raman spectroscope operated with an 830 nm Raman laser at 125 mW. A total of 1,005 spectral data points in the Raman spectrum, spanning from 400 to 1700 cm^-1^, were recorded. The spectrum from polystyrene was recorded to for each trail calibrate the Raman shift and signal intensity. Leaf discs were excised using a hole puncher and placed on the sample stage of the Raman spectroscopy system. Five Raman spectra were acquired from a single spot: three different spots on one leaf disc per leaf and two leaves per plant from 3 to 5 plants. Therefore, for each treatment or inoculation, a total of 90 to 150 spectra were acquired, with an acquisition time of 10 seconds per spectrum. A modified version of the previously described Elicitor Response Index (ERI) and Infection Response Index (IRI) (Chung et al., 2021) was used to quantify PTI in plants. Briefly, the difference and significance (*p<0.05*) between the mean spectrum of the experimental group (elicitor or pathogen treatment) and that of the control group (mock treatment) were calculated. The difference could be either positive or negative. The ERI or IRI is defined as the area under the curve of the significant spectral differences. Calculations were performed in MATLAB using the following script:


[hval,pval]=ttest(sample1, sample2);

newPval=mafdr(pval);

indexPval=newPval(1,:)<0.05;

meandiff=sample1mean−sample2mean;

ERI=sum(meandiff.∗indexPval);

IRI=sum(meandiff.∗indexPval)

## 3 Results

### 3.1 Chitin treatment specifically induced a significant Raman shift

**Figure 1:**
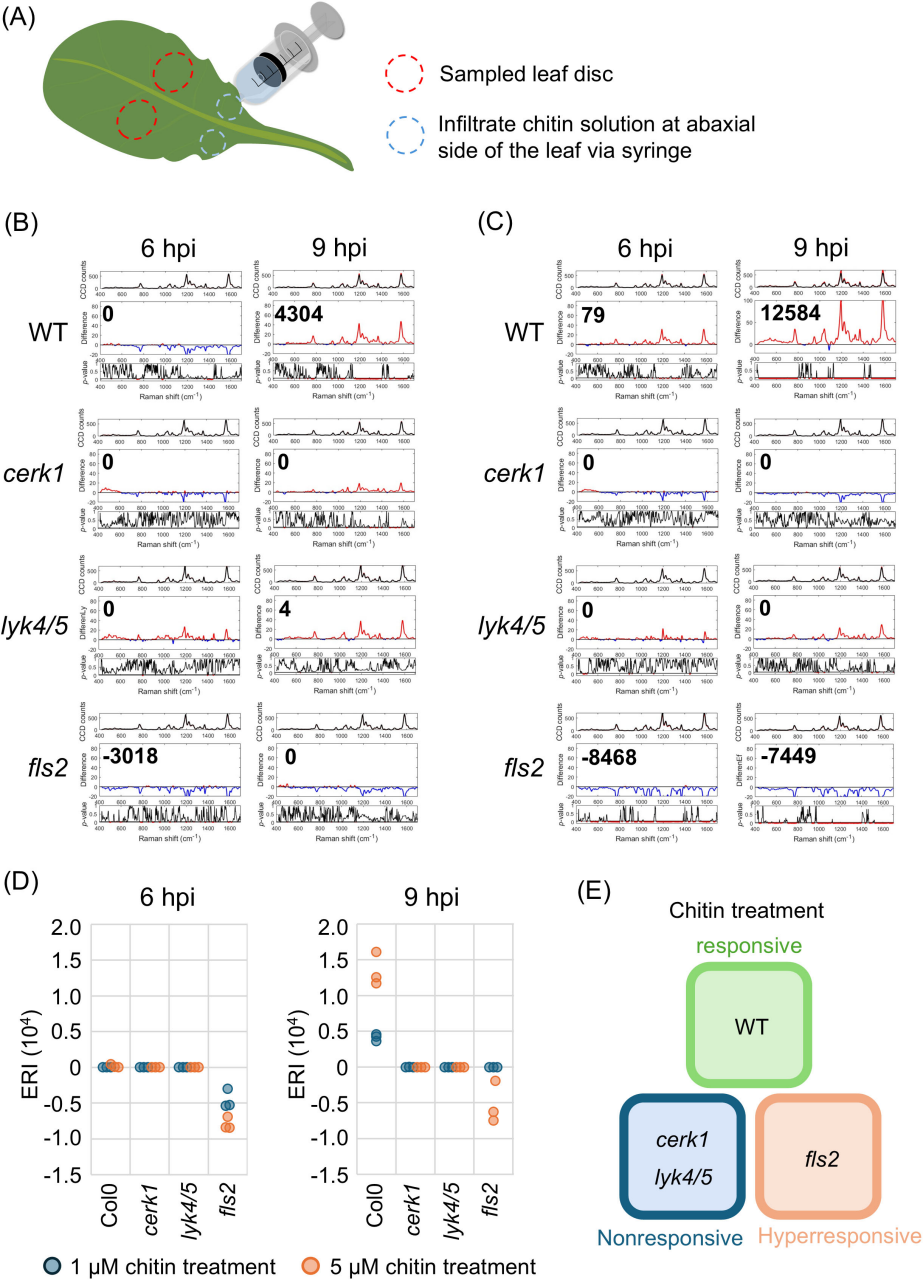
The detection of PTI induced by chitin treatment. The leaves of four-week-old wild type (Col-0, WT) and receptor mutans (*cerk1, lyk4/5* and *fls2*) were infiltrated with chitin solution. The ERI was acquired at 6 and 9 hours-post-infiltration (hpi), respectively. **(A)** A schematic diagram depicting the process of chitin treatment and sampling. The chitin emulsion was infiltrated at abaxial side of 7^th^ and 8^th^ leaves via syringe. The leaf disc next to the infiltration site was collected for analysis. The analytical results using Raman spectroscopy from the leaves infiltrated with **(B)** 1 µM, or **(C)** 5 µM chitin emulsion were presented. A group of three sub-panels represented the results from 3 to 5 biological independent replicates. Upper panel: the average of 36 to 60 spectra from mock- (black) and chitin- (red) treated leaves; Middle panel: the difference from two spectra shown in upper panel, red and blue segments indicate positive and negative value, respectively. The ERI (Elicitor Response Index) was shown at the top left. Lower panel: t-test was used to evaluate statistical significance of the differences. Red and black segments indicate p-value lower or higher than 0.05, respectively. **(D)** The ERI from triplicated trials in the respective lines treated with solutions containing 1 or 5 µM chitin. **(E)** The illustration describing the sensitivity of each line to chitin treatment.

As a well-known fungal PAMP treatment with chitin elicits plant responses mimicking those obtained with fungal infection; therefore, chitin can be used as a surrogate to characterize Raman shift specifically associated with fungal infection. We infiltrated four-week-old Arabidopsis with chitin emulsions at concentrations of 1 or 5µM. Chitin solutions were infiltrated via needle-less syringe on the abaxial side of leaf and leaf discs were sampled for analysis by using Raman spectroscopy (**Figure 1A**). The Raman spectra were acquired using a custom-built Raman spectrometer equipped with an 830 nm infrared laser (**Supplementary Figure 1A**). To minimize biological variance among individuals and enhance the spectral differences induced by chitin, we followed the method developed by Chung et al. (2021) and calculated the mean difference and *p*-value between the spectra from chitin-treated and mock-treated leaves, each comprising 18 to 30 spectra (**Supplementary Figures 1B, C**). This analysis led to the generation of the Elicitor Response Index (ERI (Chung et al., 2021)). We found that wild type plants (WT, Col-0) began to exhibit a positive ERI at 9 hours post-infiltration (hpi, **Figures 1B, C**). The ERI ranged from 3,631 to 4,591 with 1 µM chitin and from 11,758 to 16,107 with 5 µM chitin, indicating a dose-dependent chitin sensing system (**Supplementary Figures 2B, D**). The ERI from WT plants usually peaked at around 9 hpi which was then followed by a decrease from 12 to 24 hpi (**Supplementary Figure 3**). Chitin receptor mutants, *cerk1* and *lyk4/5*, were used to verify the specificity of this response. Although some trials showed a positive difference (red line, middle subplot), the corresponding *p*-values did not meet the significance level of<0.05, resulting in an ERI of 0. Overall, both mutants exhibited an ERI of 0 or close to 0 at 3, 6, and 9 hpi (**Figure 1**, **Supplementary Figures 2**, **4A**) confirming their insensitivity to the chitin-induced signaling pathway. These results suggest that CERK1 and Lys4/5 are not only the primary receptors to activate downstream signaling pathway but also critical for triggering Raman shift.

In Arabidopsis the FLS2 receptor is involved in sensing bacterial infection. To investigate the specificity of various pathogen receptors, we examined the effects of chitin treatment on *fls2* mutant. Unexpectedly, *fls2* exhibited ERI values ranging from 0 to negative, rather than the expected positive responses, despite having an intact chitin-sensing systems (**Figures 1B–D**). Higher concentrations of chitin appeared to result in reduced ERI values, indicating an inverse dose-dependent correlation between chitin concentration and ERI. Further analyses revealed a positive ERI at 3 hpi in *fls2* plants; in contrast the ERI peaked at 9 hpi in WT plants (**Supplementary Figure 4B**).

The differences between *fls2* and chitin receptor mutants were also reflected in the phosphorylation of MAPK (**Supplementary Figure 5**). A typical phosphorylation of MPK6, MPK3, and MPK4/11 was detected 5 to 10 minutes after treatment with 5 µM chitin. In *cerk1* and *lyk4/5* mutants, the accumulation of phosphorylated MPKs was significantly lower and diminished more rapidly. By contrast, phosphorylation levels in *fls2* were not as pronounced as those in WT but the responses persisted longer.

In comparison to the insensitive *cerk1* and *lyk4/5* mutants, the observed negative shift difference in *fls2* may indicate a hyper-responsive state characterized by an early decrease in pigment content (**Figure 1D**). This hyper-responsive status appears to correlate with the prolonged MAPK activation in this study and may serve as a distinguishing feature between receptors belonging to the two distinct Pattern-Triggered Immunity (PTI) systems.

### 3.2 Fungal inoculations induced significant IRI on Arabidopsis

**Figure 2:**
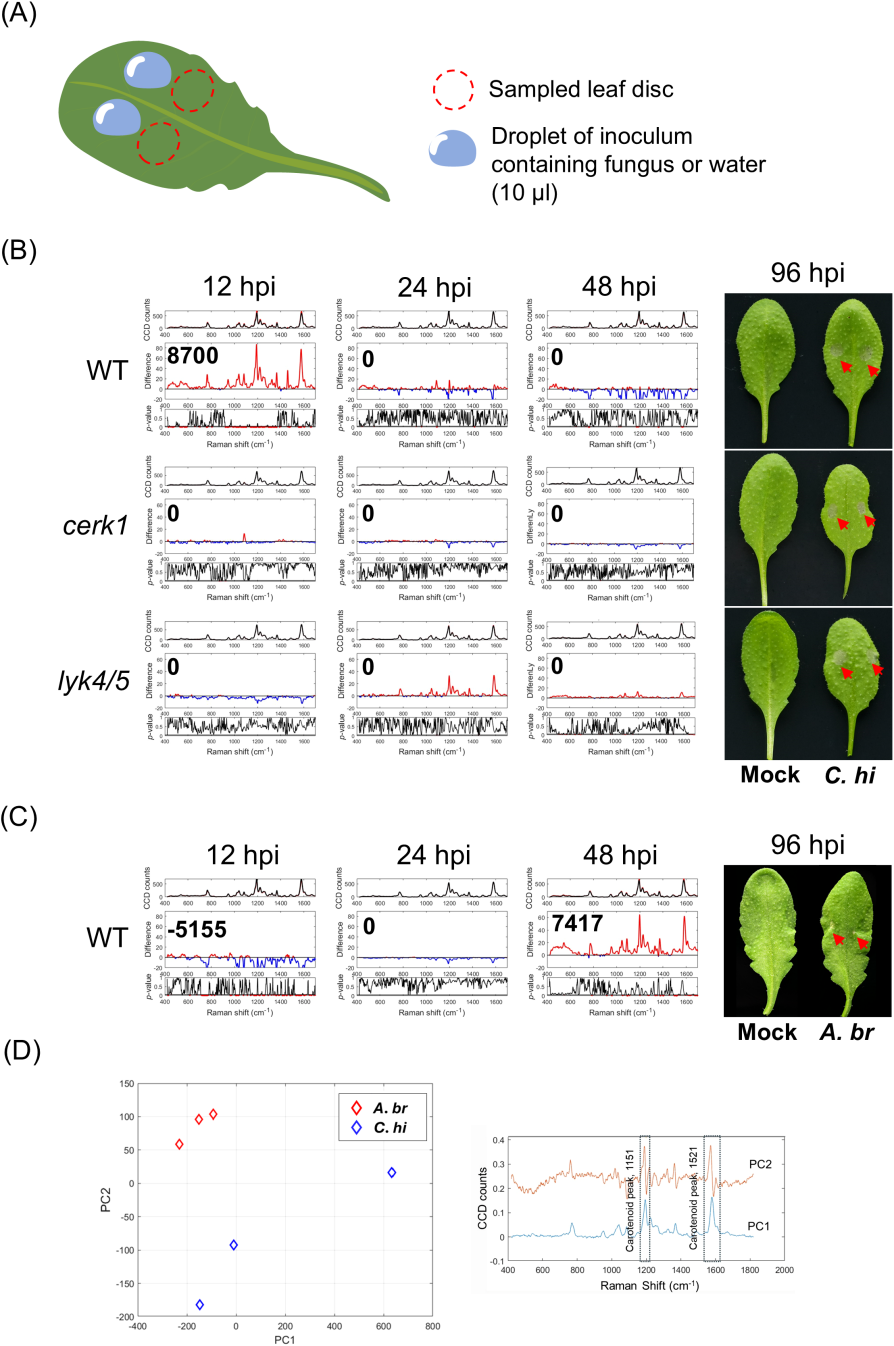
Detection of fungal infection on Arabidopsis using Raman spectroscope. **(A)** A schematic diagram depicting the process of fungal inoculation and sampling. Three-week-old plants of Arabidopsis were used for the experiments. An inoculum droplet containing fungal spores with the indicated concentration was deposited on two spots on a leaf blade (leaf 7 and 8) as indicated. The leaf disc from the region next to the inoculated site (red dotted circle) was collected for the Raman spectral analysis. **(B)** Plants were inoculated with *Colletotrichum higginsianum* (5 × 10^6^ spores/mL). **(C)** Plants were inoculated with *Alternaria brassicicola* (10^7^ spores/mL). The Raman spectra acquired at 12, 24 and 48 hpi were shown along with the corresponding Infection Response Index (IRI). The phenotypes of mock- and fungus-inoculated leaves at 96 hpi were shown at the right panels (red arrows). Each IRI represents the results from 3 to 5 biological replicates which comprise 36 to 60 spectra from mock- and fugus-inoculated leaves. **(D)** Principal component analysis (PCA, left panel) method to distinguish the two different fungal infections at 48 hours. The plot displays PC1 and PC2 spectra (right panel), with a dashed box highlighting key spectral features such as the carotenoid peak. Mock, mock-treated leaf; C.hi, *C. higginsianum* inoculated leaves; A.br, *A. brassicicola* inoculated leaves.

Next, to see if a similar Raman shift could be detected with fungal infections, we inoculated WT Arabidopsis plants with *Colletotrichum higginsianum* or *Alternaria brassicicola*. *A. brassicicola* is one of the most notorious fungi that infect *Brassicaceae* which includes many economically important crops (Nowicki et al., 2012). *C. higginsianum* which has a wide host range can cause anthracnose disease leading to severe economic loss (Kleemann et al., 2012). To facilitate symptom observation, we used three-week-old Arabidopsis plants for the trials. We aimed to observe Raman shifts induced by fungal propagation instead of chitin from cell wall of fungus in the inoculum. Therefore, to minimize this potential false-positive effect, we collected leaf discs approximately 2 mm away from the inoculation site at 12, 24, and 48 hpi for RS analysis (**Figure 2A**). We found that inoculation with *C. higginsianum* on WT plants induced positive IRI at 12 to 24 hpi (**Figure 2B**, **Supplementary Figure 6A**). By contrast, inoculation of *cerk1* and *lyk4/5* mutant plants did not generate positive IRI throughout the experimental period (**Figure 2B**, up to 48 hours). Notably, symptoms in both WT plants and receptor mutant plants primarily appeared from 96 hpi onwards. However, more severe symptoms, such as hampered leaf growth and thoroughly necrotic lesions could be found in mutant leaves as a result of the compromised sentinel system (Huaping et al., 2017; Miya et al., 2007; Wan et al., 2008). Our results also indicated that the metabolic fluctuation recognized in the Raman shift was highly correlated with the PTI response.

In contrast to *C. higginsianum* infection, *A. brassicicola* infection generally produced negative IRI at 12 hpi, transitioning to positive IRI at 48 hpi whereas the corresponding symptoms typically manifested at 96 to 120 hpi (**Figure 2C**, **Supplementary Figure 6B**). The inoculation of either fungus potentially led to a negative IRI at 72 hpi (**Supplementary Figure 6C**). These results suggest that IRI peak may appear at different time points due to potential biological differences amongst trials. However, positive IRI generally appeared 2 to 3 days prior to the observable symptoms, indicating its potential as an early marker of fungal infection.

We also analyzed the spectral data using principal component analysis (PCA) to see if we could differentiate the two different fungal inoculations on Arabidopsis. The PC1 versus PC2 plot (**Figure 2D**, left panel) at 48 hpi showed a clear separation between the two different fungal inoculations i.e. *C. higginsianum* and *A. brassicicola*. The clear separation between the fungi inoculations confirms that Raman spectra were able to effectively differentiate infections by the two fungi. The plot presents the PCA loadings for PC1 and PC2, with dashed box highlighting key spectral features like carotenoid peaks at 1151 cm^-1^ and 1521 cm^-1^ (Gupta et al., 2020; Movasaghi et al., 2007), suggesting their role as important stress makers (**Figure 2D**, right panel).

### 3.3 Fungal inoculations induced significant IRI in *Brassica* vegetables

**Figure 3:**
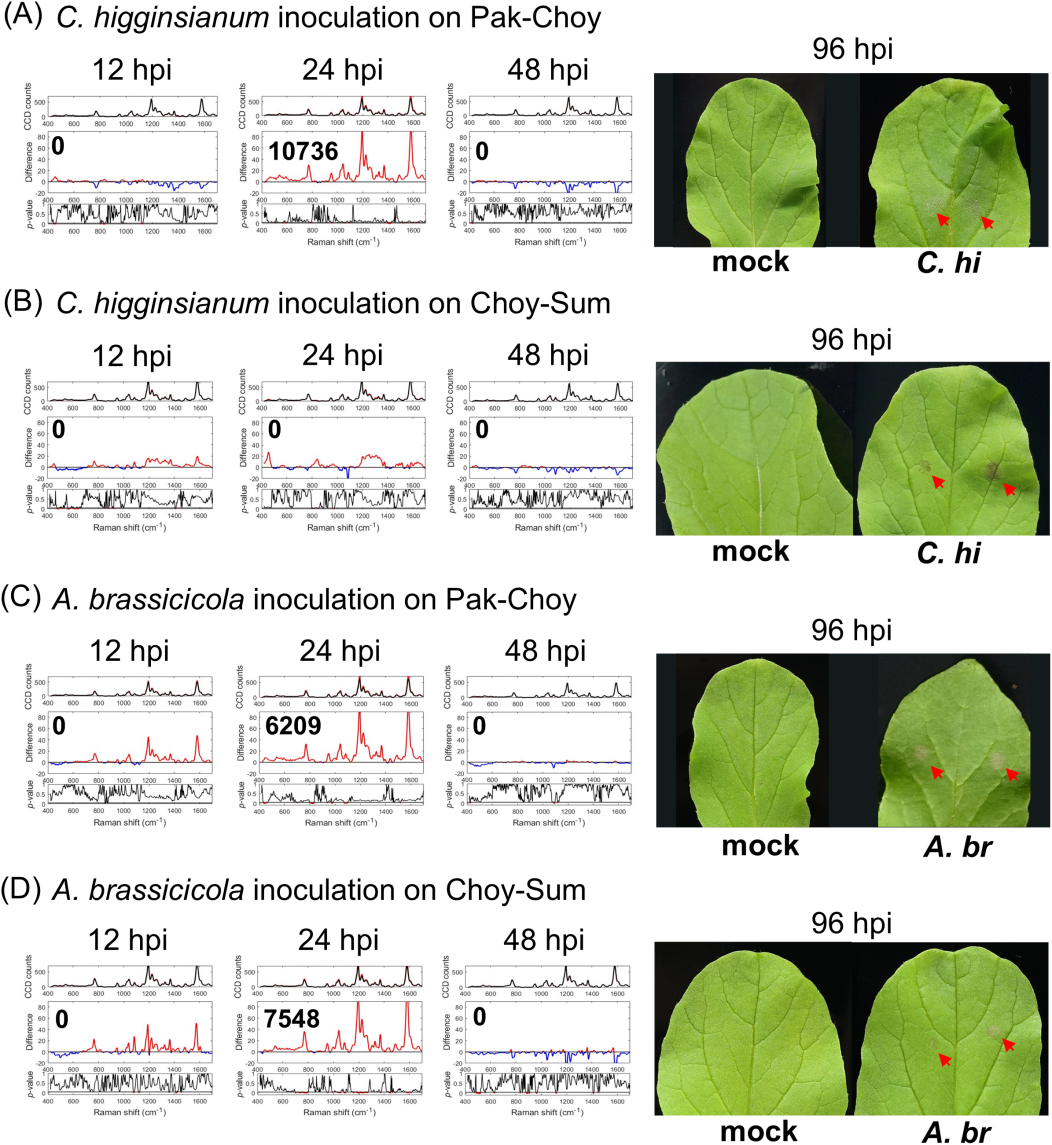
Raman spectroscopic analysis of *Brassica* spp. infected with fungi. Sixteen- to eighteen-day-old plants of two types of vegetables, Pak-Choy (*Brassica rapa* subsp. *chinensis*) and Choy-Sum (*Brassica chinensis* var. *parachinensis*), were used. Leaf 3 and leaf 4 were inoculated with *C. higginsianum* (5 × 10^6^ spores/mL) **(A, B)** or with *A. brassicicola* (10^7^ spores/mL) **(C, D)** with the method described in **Figure 2A**. Leaf discs were collected at 12, 24, 48 hpi for analyses by Raman spectroscopy. Each IRI represents the meaning of 3 biological replicates. Right panels show leaf symptoms at 96 hpi (red arrows). *C. hi*, *C. higginsianum* inoculated leaf; *A. br*, *A. brassicicola* inoculated leaf.

To evaluate the effectiveness of RS in detecting early fungal infections in crops, sixteen- to eighteen-day-old Pak-Choy and Choy-Sum were subjected to *C. higginsianum* inoculation assay as described above. At this stage, plant sizes of Pak-Choy were smaller than those of Choy-Sum; the third and fourth leaves of these two vegetables were used for fungal inoculations. We found that infected Pak-Choy plants exhibited a positive IRI at 12 to 24 hpi (**Figure 3A**) or at both 12 and 24 hpi (**Supplementary Figure 7A**, Trial 2). By contrast, infected Choy-Sum plants showed only minor fluctuations in Raman shift and did not display any positive IRI within 48 hpi (**Figure 3B**, **Supplementary Figure 7B**). These findings suggested that Pak-Choy and Choy-Sum may exhibit different levels of perceptiveness to *C. higginsianum*. To further substantiate these results, we conducted similar trials with *A. brassicicola*. For both vegetable varieties a positive IRI primarily appeared at 24 or 48 hpi, whereas noticeable symptoms typically emerged between 96 to 120 hpi (**Figures 3C, D**, **Supplementary Figure 8**). Notably, although the positive IRI appeared at a particular time point, a positive spectral difference was seen during much of the post-infection monitoring period, (**Figure 3**, **Supplementary Figures 7**, **8**). This suggests that the signal induction period is prolonged in the vegetable crops allowing a broader detection window for diagnosis.

### 3.4 Evaluate the detection performance in randomized controlled trials

To evaluate the effectiveness of this method for detecting fungal infection in Arabidopsis, we tested its applicability with a randomized control trial. As *A. brassicicola* infection consistently generated positive IRI at 48 hpi (**Figure 2C**, **Supplementary Figure 6B**), we randomly inoculated 22 WT Arabidopsis plants with *A. brassicicola* whereas 20 WT plants were inoculated with water serving as mock controls. Leaf samples collected at 48 hours post-inoculation (hpi) were analyzed using RS. All spectra acquired were compared to a standard mock-inoculated spectrum obtained from 3 independent plants.

**Figure 4:**
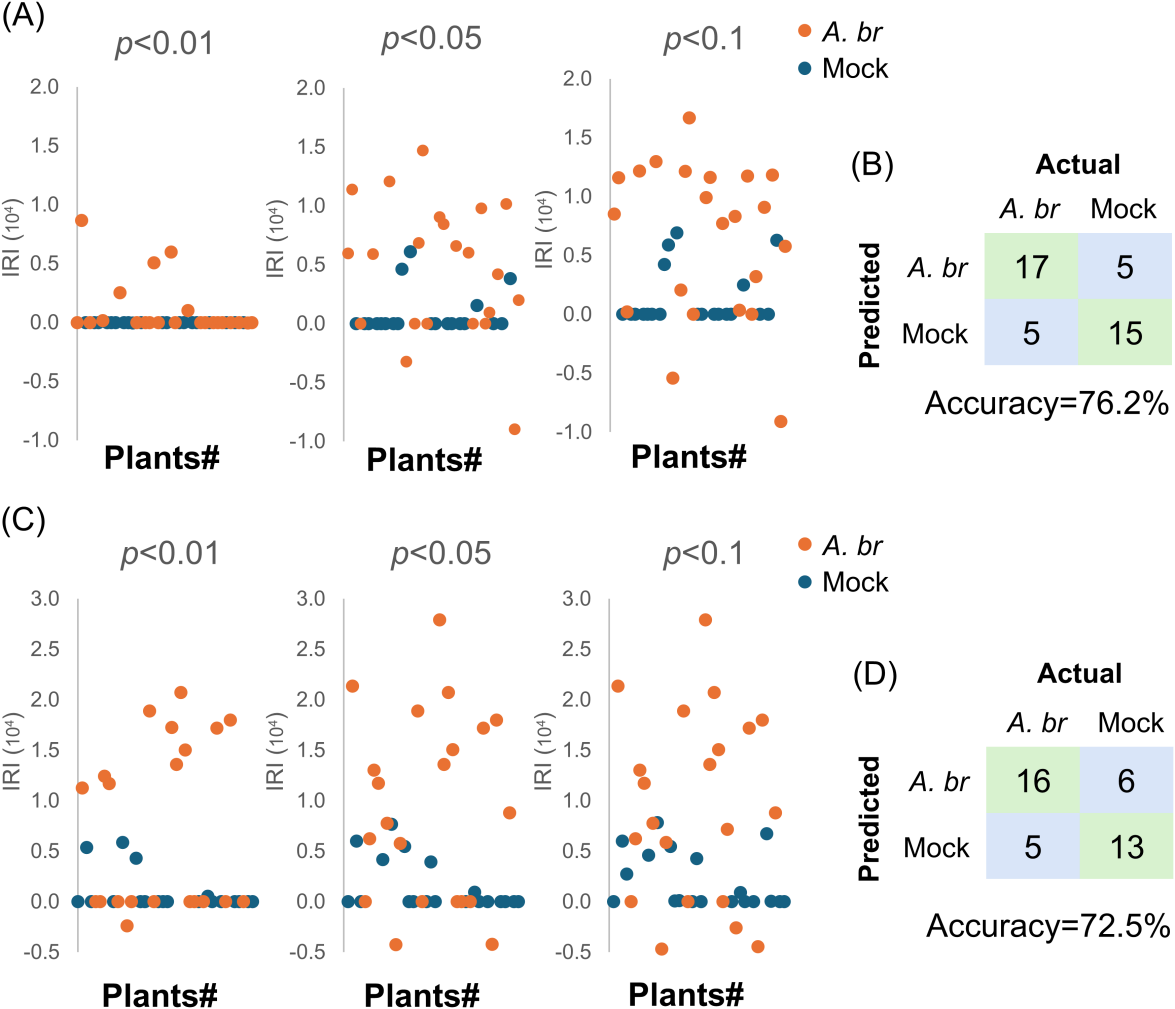
Detection of potential fungal infection on Arabidopsis or Pak-Choy by Raman spectroscopic analysis in a randomized controlled trial. A total of 22 and 20 WT Arabidopsis plants **(A, B)**, or a total of 21 and 19 Pak-Choy plants **(C, D)** were inoculated with *A. brassicicola* (*A.br*) or water (mock), respectively. The mean spectrum from each plant was compared to that from an independent trial comprising three standard mock-treated plants. **(A, C)** The IRI of each plant was calculated at a significance level of *p*<0.01, *p*<0.05 and *p*<0.1 in t-test and shown, respectively. **(B, D)** Confusion matrix showing the performance of the diagnostic method. Symptoms on the inoculated leaves at 120 hpi.

We found that only 6 out of 22 inoculated plants exhibited positive IRI at a significance level of *p<0.01*. At the *p<0.05* level, 15 inoculated plants displayed positive IRI and 2 inoculated plants displayed negative IRI (**Figure 4A**). Additionally, 4 out of 20 mock-inoculated plants were also displayed positive IRI. When evaluating IRI with a threshold of *p<0.1*, 18 inoculated plants with positive IRI remained identifiable; however, 5 mock-inoculated plants produced false positives. There was no visible symptom on all mock-inoculated plants at 120 hpi, despite some of them having shown positive IRI, such as plant #16 (**Supplementary Figure 9A**). No obvious differences on the symptom severity could be observed among the inoculated plants with different IRI values (**Supplementary Figure 9A**, #19, #31 and #12). Considering that the PTI response may appear as a negative spectral difference (**Supplementary Figure 6C**), two plants exhibiting negative IRI were classified as infected. In total, 17 out of 22 plants were identified as true positives, whereas15 out of 20 plants were classified as true negatives, resulting in an accuracy rate of 76.2% (32/42) when a threshold of *p<0.05* was applied (**Figure 4B**).

We further investigated the applicability of this method on Pak-Choy with a similar trial. A total of 40 Pak-Choy plants were included, with 21 inoculated with *A. brassicicola* and 19 receiving a mock treatment. Leaf discs collected at 24 hpi were analysed by RS (**Figure 4C**). Among the fungus-inoculated plants, 10 out of 21 exhibited positive IRI results at *p*<0.01. Additionally, 14 or 15 fungus-inoculated plants displayed positive IRI values at *p*<0.05 or *p*<0.1, while most of the mock-inoculated plants had IRI values around 0. In the mock-inoculated group, none of the plants displayed visible symptoms at 120 hpi (**Supplementary Figure 9B**), as shown by the leaves from plant #9 (IRI = 0) and plant #11 (IRI = 7660). Among the fungus-inoculated plants, plant #5 had an IRI of 0, whereas plant #12 recorded the lowest IRI of -4280. Notably, both plants exhibited similar symptom severity to plant #22, which had the highest ERI of 27890 (**Supplementary Figure 9B**). Including the two plants that exhibited negative IRI values at *p*<0.05, 16 out of 21 fungus-inoculated plants were identified as true positives, whereas 13 out of 19 mock-treated plants were identified as true negatives (**Figure 4D**). This collectively yielded an accuracy of 72.5% (29 out of 40). Overall, these results show that fungal infections in vegetables can be detected by RS prior to the onset of visible symptoms.

### 3.5 Specific Raman features associated with bacterial and fungal infections

As practical strategies for combating fungal and bacterial infections differ significantly accurate identification of these threats is essential for implementing appropriate measures against pathogen infections. To acquire Raman spectrum induced by bacterial infection or the bacterial PAMPs, WT leaves were inoculated with *Pseudomonas syringae* DC3000 or 1 µM flg22 and analysed by Raman spectroscopy as previously described (Chung et al., 2021). Positive IRI from DC3000 inoculation primarily appeared at 24 dpi; whereas the ERI from flg22 treated leaves showed a positive value at 9 hpi (**Supplementary Figures 10A, B**).

PCA of the spectral data revealed distinct differences between fungal inoculations and bacterial infection (**Supplementary Figures 10C, D**). The PC1 *vs*. PC2 plots at 12, 24, and 48 hpi demonstrated a clear separation between the two fungal inoculations and bacterial infection (DC3000), confirming that Raman spectroscopy effectively distinguishes these treatments. Key Raman spectral peaks, including pectin (742.3 cm^-1^) and carotenoids [1151 cm^-1^ (Gupta et al., 2020) and 1521 cm^-1^ (Movasaghi et al., 2007)], contributed significantly to PC1 and PC2, indicating their significance as stress markers (**Supplementary Figure 10C**). Additionally, **Supplementary Figure 10D** presents the PC1 *vs*. PC2 plot for flg22 and 5 µM chitin at 9 hpi, further highlighting the ability of PCA to differentiate plant responses to distinct elicitor treatments (**Supplementary Figure 10D**).

**Figure 5:**
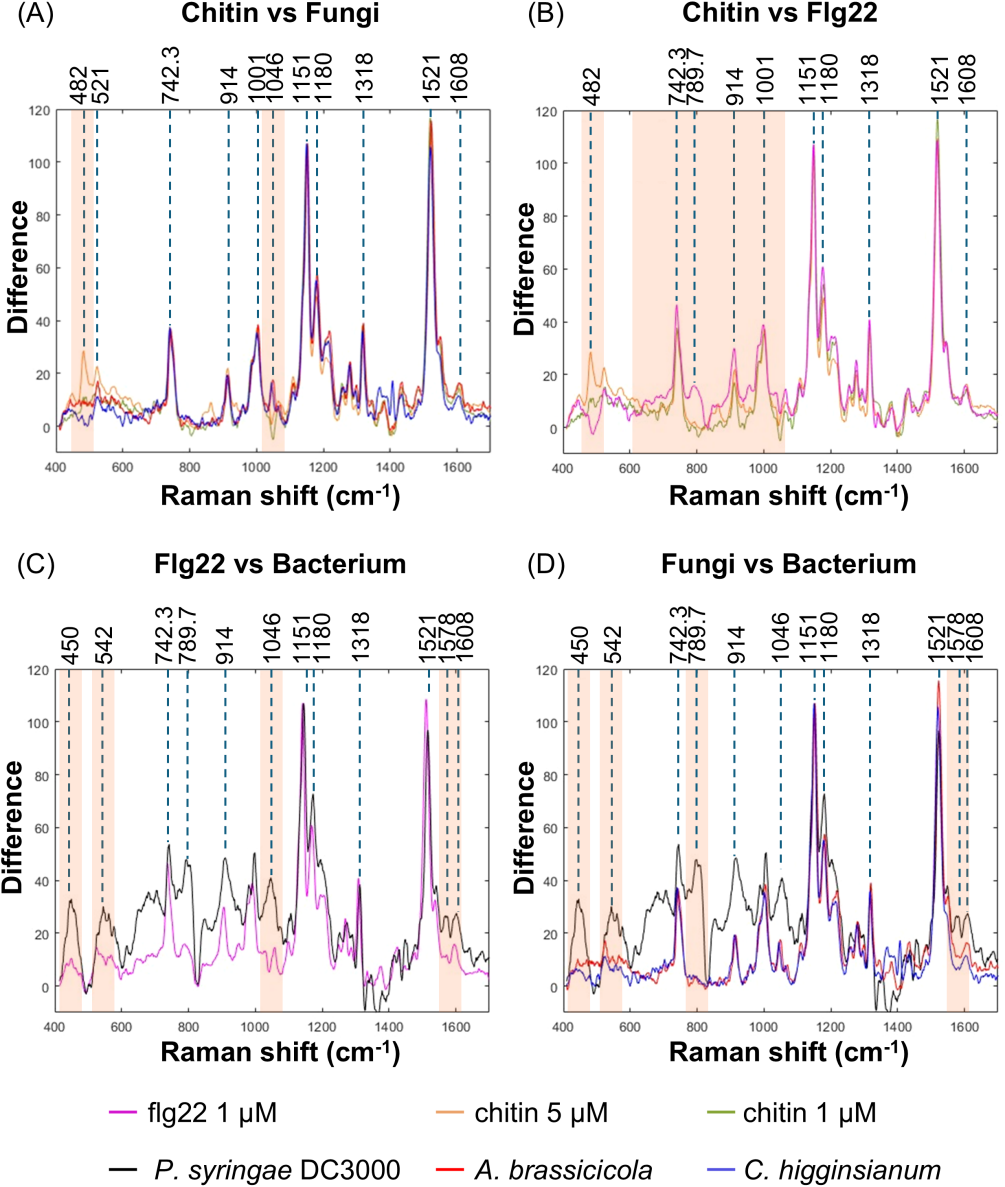
Raman spectral differences between bacterial and fungal infection. Raman spectra from WT Arabidopsis leaves treated with different elicitors or inoculated with different pathogens were overlapped for the comparison. The spectral intensity was calibrated by the carotenoid peak at 1151 cm^-1^ from Flg22 treated leaves. The spectrum from each treatment or inoculation represents the average data from three independent experiments. The spectral overlapping from chitin treatment and fungal infection **(A)**, chitin and Flg22 treatment **(B)**, Flg22 treatment and bacterial infection **(C)**, as well as fungal and bacterial infection **(D)** were presented. Orange shadow indicated the featured band in respective comparison.

We overlapped the spectra from chitin and flg22 treatments at 9 hpi. The intensity of the respective spectrum was normalized by the carotenoid peak at 1151 cm^-1^ from flg22 treatment (**Figure 5**). We found that the spectra from treatments with 1 and 5 µM chitin exhibited similar signatures, with only minor variances in peak intensity at 482 and 521 cm^-1^ (**Figure 5A**, **Supplementary Figure 11A**). This suggests that the Raman spectrum signatures are consistent and not influenced by dose-dependent factors. The spectra from the time point showing the highest IRI after fungal inoculation were used for the same comparison. The results indicated that the infection of the two fungi exhibit similar spectral features, with the exception of the peak at 482 cm^-1^, which is present only in chitin-treated leaves. Additionally, the peak at 1046 cm^-1^ increased in fungal-infected leaves but decreased in chitin-treated leaves (**Figure 5A**, **Supplementary Figure 11A**).

By contrast, the spectrum from flg22 treatment displayed a typical peak at 789.7 cm^-1^, which is associated with the vibration of phosphodiester bond (**Table 1**), when compared to that from chitin treatment (**Figure 5B**, **Supplementary Figure 11B**). Moreover, there were some differentiated features, such as increased intensity in the wave number region less than 1001 cm^-1^. Similar but more drastic fluctuations could be observed in the spectrum from *P. syringae* DC3000 inoculated leaves. Note the distinct peaks at 542, 789.7, 1046, and 1578 cm^-1^ (**Figure 5C**, **Supplementary Figure 11C**).

**Table 1:** Significant vibrational bands and their assignments that contribute to the high ERI or IRI value.

Raman peak (cm^-1^)	Assignment	Vibrational mode	Reference
542	Flavonoids	C=C stretching rin B + C=C bending ring A and vinyl group	Moreno et al., 2022
742.3	Pectin	*γ*(C-O-H) of COOH	Gupta et al., 2020
789.7	phosphodiester bands in DNA	O-P-O stretching	Movasaghi et al., 2007
1001	Carotenoids	ν3 (C–CH_3_ stretching), phenylalanine ring stretching mode	Gupta et al., 2020
1046	Nitrate	NO_3_ stretching	Gupta et al., 2020
1151	Carotenoids	C–C stretching; v(C–O–C)	Gupta et al., 2020
1180	Carotenoids	dCH, u (pyr half-ring)_as_	Perez-Guaita et al., 2017
1318	Cellulose/Lignin /Protein	dCH_2_ bending	Gupta et al., 2020
1521	Carotenoids	–C=C– (in-plane)	Movasaghi et al., 2007
1578	Quinonoids	C=C stretching	Moreno et al., 2022
1608	Lignin	ʋ (C–C) aromatic ring +δ(CH)	Gupta et al., 2020

Through the overlapping of spectra from *P. syringae* DC3000, *A. brassicicola*, and *C. higginsianum*, as well as elicitor treatments, similar differences between fungal and bacterial infection could be easily distinguished. Note that the peak at 1046 cm^-1^ associated with nitrate was found only in the pathogen inoculations but not the elicitor treatments (**Figure 5D**, **Supplementary Figure 11D**).

## 4 Discussion

Our previous study has demonstrated the application of RS for detecting early bacterial infections in plants (Chung et al., 2021). We introduced the Elicitor Response Index (ERI) and Infection Response Index (IRI) to quantify significant increase in spectral intensity and evaluate PTI response in inoculated leaves. In this previous study, both indexes competently identified PTI response at 24 hpi following elicitor treatment or pathogen introduction, however, the decrease in spectral intensity that often followed the appearance of the ERI or IRI peak was not considered. Our results here, along with previous observations, suggest that the negative difference should be also considered as a feature in the responses to pathogens. Therefore, in this study, we employed a modified ERI/IRI which could represent both significantly positive or negative differences in Raman spectra. Our results showed that following chitin treatment, WT Arabidopsis showed a positive ERI that peaked at 9 hours post-inoculation (hpi) and subsequently decreased from 12 hpi onward. (**Figure 1**, **Supplementary Figures 2**, **3**). To verify the response specificity, we performed similar assays with chitin receptor mutants, *cerk1* and *lyk4/5* which displayed ERI of 0 or close to 0 when treated with chitin or inoculated with fungi (**Figure 1**, **Supplementary Figure 2**). The mutants *cerk1* or *lyk4/5* were previously reported to show increased susceptibility to *Alternaria brassicicola*, *Erysiphe cichoracearum* and *Fusarium oxysporum* f. sp. *cubense 4* (Huaping et al., 2017; Miya et al., 2007; Wan et al., 2008) and similar results were observed following inoculation with *C. higginsianum* (**Figure 2B**). Infected leaves of *cerk1* and *lyk4/5* showed lesions of smaller sizes but more severe water-soaked leaves as compared to infected WT leaves. Notably, chitin treatment on the knock-out (KO) mutants of bacterial pattern recognition receptors (PRRs), *fls2* resulted in 0 to negative ERI at 6 and 9 hpi, rather than the anticipated positive ERI observed in WT. Further analyses revealed a positive spectral fluctuation at 3 hpi (**Supplementary Figure 4**), Consistent with these findings, *fls2* demonstrated weaker yet a more prolonged MAPK activation when compared to WT (**Supplementary Figure 5**).

Chitin and its derivative, chitosan are known for their ability to boost plant immune responses across a wide range of plant species. These elicitors serve as a signal to activate plant immune systems. The recognition of chitin by its receptors prompts a series of defensive responses, including the production of reactive oxygen species, the activation of signaling pathways, and the expression of defence-related genes. These responses not only provide direct antimicrobial effects but also endow the treated plants with defenses against future pathogen attacks. The ability of chitin to universally trigger such immune responses renders it a crucial component in the enhancement of plant resilience to pathogens like bacteria, fungi, viruses, and nematodes (Saberi Riseh et al., 2024).

In addition to its direct antimicrobial effects that inhibit the proliferation of various microorganisms, an increasing number of studies have also shed light on the crosstalk between bacteria- and fungi-induced immunity. A recent study has indicated that the common regulatory kinase BAK1 can phosphorylate the juxtamembrane (JM) region of CERK1 in response to treatment with pathogenic bacteria or bacteria-derived PAMPs, such as flg22 and elf18. While this bacteria-mediated phosphorylation does not fully phosphorylate CERK1 as observed with chitin treatment, the findings suggest that it primes CERK1 to enhance its signaling capabilities in response to chitin. Consequently, this priming leads to increased resistance against potential fungal infections (Gong et al., 2019). On the other hand, chitin soil amendment was reported to trigger systemic resistance by enhancing PTI against *P. syringae* infection in lettuce, tomato and Arabidopsis (Makechemu et al., 2024). Micrografting experiments confirmed that chitin perception in roots strengthens the expression of key PTI components in distal leaves, including BIK1 and RBOHD. Chitin addition also renders plants more responsive to subsequent treatment of flg22 or elf18 resulting in a stronger ROS burst and potentiated calcium influx (Makechemu et al., 2024). Despite these differences, the significant crosstalk between the fungal and bacterial MAPK pathways leads to a more robust and coordinated defence response. Common components within the MAPK signaling cascades could integrate signals from both types of pathogens, allowing plants to generate a typical defence mechanism via a potential mutual regulation.

The absence of FLS2 during chitin treatment may potentially disrupt the homeostasis maintained by the intrinsic crosstalk between the two PTI systems. The positive spectral difference observed as early as 3 hours post-inoculation (hpi) suggests a hyper-responsive state that precedes the expected recession seen in WT plants after 12 hpi (**Supplementary Figures 3**, **4**). This finding may also indicate the possibility of an ultrasensitive response caused by multisite phosphorylation or potential allosteric regulation from FLS2-mediated PTI (Ferrell and Ha, 2014a, b). It is conceivable that a faster, albeit partial, activation of PTI could be triggered due to a lowered response threshold to the elicitor or pathogen (**Supplementary Figure 5**). Such dynamics invite further exploration into the nuances of plant immune responses and their implications for understanding plant-pathogen interactions.

Changes in carotenoids peak intensity are usually one of the most significant features to identify pathogen infection or disease severity (1151 cm^-1^ (Gupta et al., 2020), 1180 cm^-1^ (Perez-Guaita et al., 2017) and 1521 cm^-1^ (Movasaghi et al., 2007)). Carotenoids have been shown to serve not only as scavengers of reactive oxygen species (ROS) during PTI (Han et al., 2012) but also as important precursors of stress hormones, such as abscisic acid (ABA). These hormones play a crucial role in regulating the expression of downstream genes in response to both biotic and abiotic stresses (Felemban et al., 2019; Jia et al., 2022). Moreover, Raman shift can be affected by the fluctuation of metabolites content as a result of pathogenic activity, such as decomposition or fermentation (Weng et al., 2021).

The resulting differential Raman peaks could assist in distinguishing between treatments with different elicitors or infections by various pathogens. For instance, the band representing nucleic acid at 789.7 cm^-1^ increased intensity during *P. syringae* DC3000 infection and flg22 treatment (Movasaghi et al., 2007), suggesting a differential expression of defence genes. Additionally, the intensity of peaks at 482 cm^-1^, 542 cm^-1^, and the region between 742 and 1046 cm^-1^ were generally higher in comparison to both chitin treatment and fungal infection. The peaks at around 542 cm^-1^ and 742.3 cm^-1^ indicates the accumulation of flavonoids and pectin, respectively (Moreno et al., 2022; Gupta et al., 2020). These observations suggest a differential defence mechanism mediated by bacteria or their PAMPs.

A Raman band at 1578 cm^-1^ was only seen in *P. syringae* DC3000 inoculation, which may indicate increased levels of quinonoid compounds (Aymen et al., 2013), potentially derived from flavonoid metabolism. Quercetin, for example, is known to enhance resistance to *Pseudomonas syringae* pv. tomato DC3000 by modulating salicylic acid biosynthesis in Arabidopsis and this compound can be converted to various quinones via ROS-mediated pathways (An et al., 2023).

The nitrate peak at 1046 cm^-1^ generally increases in all pathogen infection scenarios. In Arabidopsis, the high-affinity nitrate transporters NRT2.1 and NRT2.2 are upregulated in response to nitrogen deficiency. Double mutants lacking both NRT2.1 and NRT2.2 exhibit significantly reduced nitrate uptake compared to WT plants, regardless of nitrogen availability. A Raman peak at 1046 cm^-1^ has been shown to decrease in intensity in nrt2.1/nrt2.2 double mutants (Huang et al., 2020). Furthermore, NRT2.1 appears to repress responses to biotrophic pathogens, potentially prioritizing resources for abiotic stress resistance over immune responses. Whereas the role of NRT2.6 in nitrate uptake is less clearly defined, its involvement in plant responses to necrotrophic pathogens seems to be related to reactive oxygen species (ROS) production and metabolic shifts, rather than a direct regulation of nitrate transport during infection. These studies emphasize the interplay between nitrate transport systems and defence signaling pathways (Camañes et al., 2012b, a; Dechorgnat et al., 2012).

Nitrate content could also be affected due to impaired nitrogen assimilation, altered nutrient allocation or compromised transport during pathogen infection. For example, *Fusarium pseudograminearum* infection was reported to reduce the ability of wheat plants to transfer nitrogen from roots to shoots (stem nitrogen transfer efficiency, or sNTE). This leads to a less efficient nitrogen use and reduced grain yield and quality (Buster et al., 2023). Infection of soybeans by *Phytophthora sojae* induces stress, causing infected plants to reallocate resources from growth towards defence mechanisms. This shift of nitrogen allocation away from growth and towards the synthesis of defence-related compounds, results in a state of apparent nitrogen limitation (Li et al., 2013). Soil-borne bacteria, fungi, and oomycetes cause vascular wilt diseases by colonizing xylem vessel. As a defence strategy against these pathogens, plants may alter xylem morphology to restrict pathogen spread but this strategy could also limit nutrient transport (Yadeta and Thomma, 2013). Collectively, the nitrate peak at 1046 cm^-1^ may serve as a marker of responses triggered by PAMPs or pathogen infection. These findings also suggest its potential application for discriminating between bacterial and fungal spectra.

In this study, we have also demonstrated the effectiveness of this RS for detecting fungal infections in both Arabidopsis and Brassica vegetables. A positive IRI was observed within 48 hpi in most experimental trials. However, Choy-Sum infected by *C. higginsianum* did not exhibit any positive IRI within the same detection window. Interestingly, the symptoms in infected Choy-Sum were more severe than those in infected Pak-Choy, and positive differences were generally noted across all three trials. A similar trend was observed in *C. higginsianum*-inoculated *cerk1* and *lyk4/5* plants (**Figures 1B, C**, **Supplementary Figure 2**), suggesting a higher susceptibility of Choy-Sum to *C. higginsianum*.

The IRI could successfully predict fungal infections in randomized controlled trials with an accuracy rate of 76.2% for Arabidopsis and 72.5% for Pak-Choy (**Figure 4**). The inoculated plants exhibited a false-negative rate of 22.7% to 23.8%, with 5% of the inoculated samples showing a negative IRI. There was no correlation between symptom severity and IRI value, indicating that the zero IRI observed should not be attributed to differential susceptibility among individuals. Considering the dynamic nature of the IRI as observed in the analyses of Raman spectra during fungal infection (**Figure 4**), our results suggest that the IRI of certain individual plants may increase at different time points. This hypothesis is supported by a parallel trial involving 30 Arabidopsis plants, which achieved an accuracy rate of 76.7% without any negative IRI (**Supplementary Figure 12**). Additionally, the false positive rate ranged from 25% to 31.6%, with instances showing no observable symptoms compared to the inoculated leaves. In summary, our results indicate that variations in metabolite content among individuals can result in differing IRI outcomes. Implementing real-time detection to monitor IRI dynamics may help mitigate this issue and enhance overall accuracy.

While Raman spectroscopy holds promise, a significant limitation is the difficulty in differentiating between closely related bacterial or fungal species by current biomarkers, such as pigments, which are often insufficient for species-level identification. Environmental factors and plant stress responses can also alter Raman spectra, potentially confounding the detection of specific pathogens (Payne and Kurouski, 2021). To better integrate data from various sources for diagnostic purposes, more work should be done in connecting Raman Spectroscopy with the knowledge graphs that model plant response to pathogens.

As highlighted by Murray et al., a systems-level approach integrating Raman spectroscopy with genomics, metabolomics, and remote sensing data is crucial for addressing the limitation (Murray et al., 2025). Plant Reactome knowledge graphs may provide insights into specific metabolite changes that are indicative of specific pathogens, enabling more targeted Raman-based detection strategies. The knowledge gaps regarding plant-pathogen interactions and the need for well-curated OMICs datasets underscore the importance of future research in this area. Large-scale data collection efforts, coupled with expert biocuration, are needed to develop robust spectral signatures for disease diagnosis.

Future research should focus on developing more specific biomarkers, integrating Raman spectroscopy data with other data types (genomics, metabolomics, remote sensing), and creating well-curated OMICs datasets to improve the accuracy and reliability of disease diagnosis. An integrative approach is essential for building robust and scalable disease surveillance systems.

## 5 Conclusion

Here, our study advances the assessment of plant defence responses against fungal infections by utilizing the Elicitor Response Index (ERI) and the Infection Response Index (IRI). Elicitor treatments and biological assays demonstrated the capability of this method to quantify metabolic changes rapidly, within hours, and to identify early responses that occur before the appearance of visible symptoms. The randomized controlled assays confirmed its applicability in predicting infections in Arabidopsis and Pak-Choy. Furthermore, the acquired spectral data show-cased the potential to distinguish plant responses to various pathogens, thereby expanding the role of RS in pathogen detection. By harnessing RS, researchers can gain valuable insights into the complex interactions between plant pigments and their functions, which enhances our understanding of plant biology and improves agricultural practices. Overall, our findings underscore the promise of RS as a non-invasive monitoring tool that can strengthen crop health management and support sustainable agricultural practices.

## Data Availability

The raw data supporting the conclusions of this article will be made available by the authors, without undue reservation.
