# Supplementary material for: Early detection of fungal infection of Arabidopsis and brassica by Raman spectroscopy
Source: Front Plant Sci. 2025 Aug 15;16:1649206. doi: 10.3389/fpls.2025.1649206 (PMC12394222; doi:10.3389/fpls.2025.1649206)
Supplement: Supplementary file 1 [file DataSheet1.docx]

**
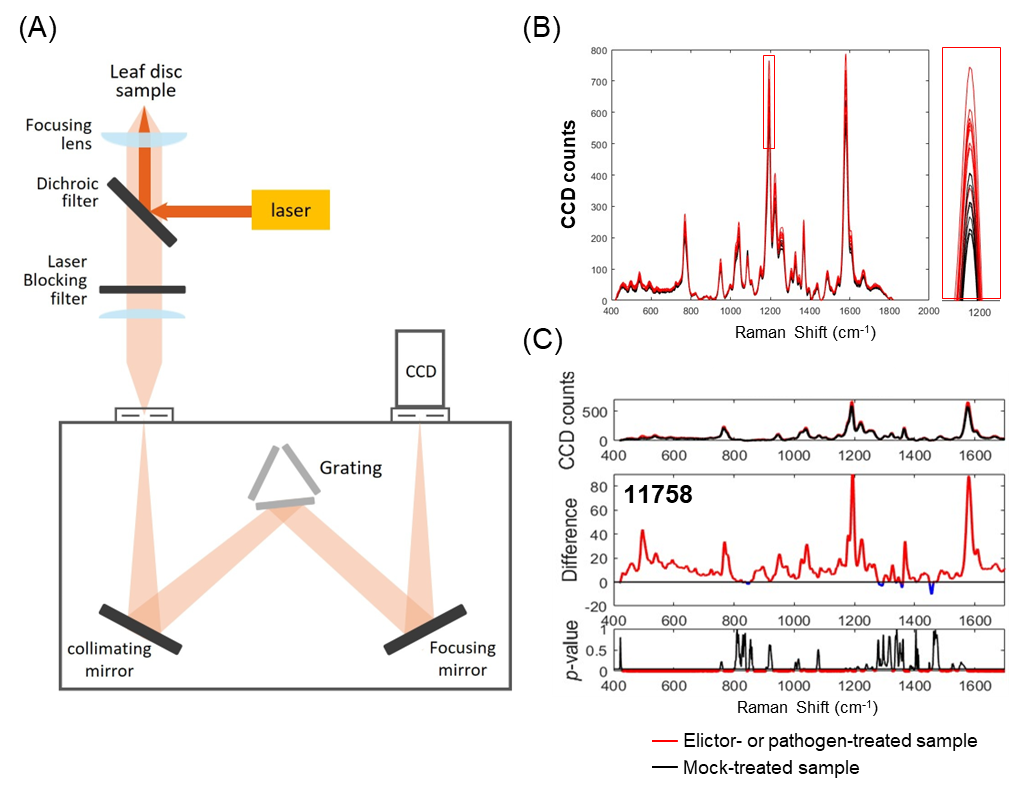
Supplementary Figure 1. Characterization of the early response induced by elicitors or pathogens through fluctuations in Raman spectra.** (A) The custom-built Raman spectroscopy is equipped with an infrared laser (830 nm) that directed towards a leaf disc sample using a dichroic filter and a focusing lens. The Raman-scattered light from the sample then passes through the dichroic filter and a laser blocking filter to remove residual laser light. Within the spectrometer, a collimating mirror directs the light towards a grating, which disperses the light into its constituent wavelengths. A focusing mirror then focuses this dispersed light onto a CCD detector, allowing for the analysis of the Raman spectrum. (B) Raw Raman spectra were collected from leaves treated with elicitors or pathogens, as well as from mock-treated leaves. The spectra were then subjected to pre-processing techniques, including cosmic ray removal, Savitzky-Golay smoothing, and polynomial background subtraction. Each curve represents the average spectrum from 3 spots on a single leaf disc. A total of 18 to 30 spectra were collected across 3 to 5 biologically independent plants, analyzing 2 leaves from each plant. (C) The upper panel displays the average spectra from elicitor- or pathogen-treated and mock-treated leaves, respectively. The middle panel illustrates the differences in spectral intensity (CCD counts), while the bottom panel presents the corresponding *p-*values, which were calculated to establish the ERI or IRI shown at the top-left of the middle panel.

**
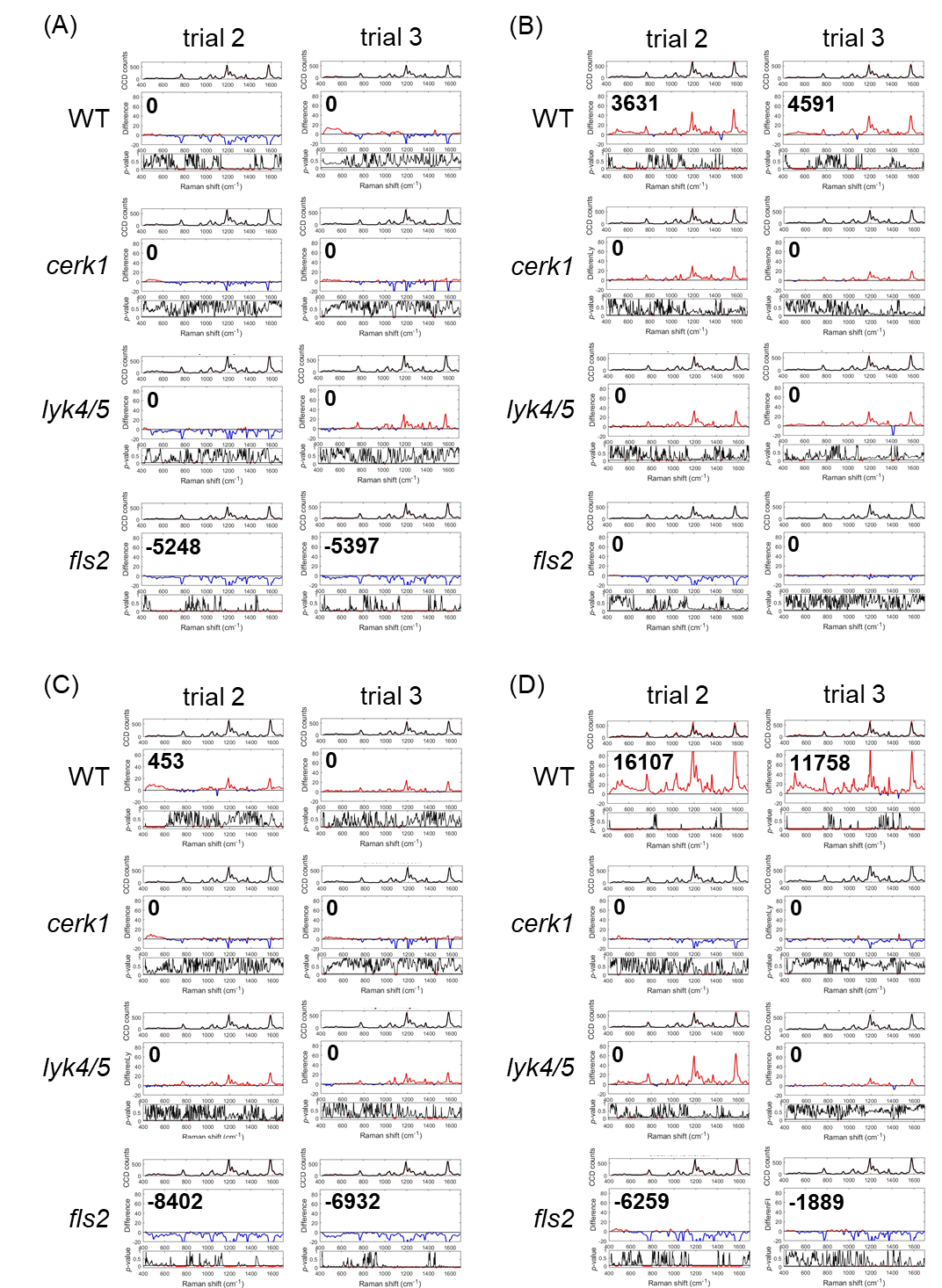
Supplementary Figure 2. Raman spectroscopic analysis of WT and various receptor mutants treated with elicitors.** Results from the other two replicated trials of Figure 1 are shown. Plants were infiltrated with 1 µM (A and B) or 5 µM (C and D) chitin solution and spectra were acquired at 6 hpi (A and C) or 9 hpi (B and D). A group of 3 panels are shown. Upper panel: a mean plot of 36 to 60 spectra from mock- (black) or chitin- (red) treated leaves; Middle panel: the difference spectrum between the mean spectrum of the elicitor-infiltrated sample and that of the H_2_O-infiltrated (mock) sample. Red indicates that the difference is a positive value, whereas blue indicates a negative value. The number inside the panel indicates the average ERI value obtained from 3-5 independent biological replicates. Lower panel: t-test was used to evaluate statistically significant differences between elicitor-infiltrated and H_2_O-infiltrated (mock) samples and the results were expressed as p-values. Red and black segments indicate *p* value lower or higher than 0.05, respectively**.**

**
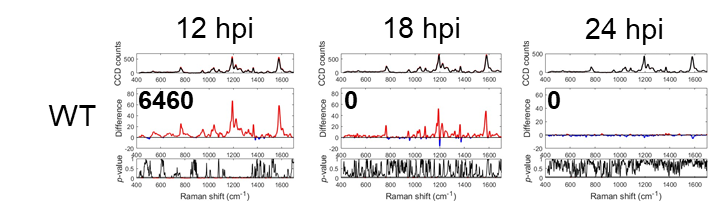
**

**Supplementary Figure 3. Chitin-induced ERI in Col-0 at 12, 18 and 24 hpi.** The Col-0 (wild type, WT) leaves were infiltrated with 5 µM chitin as described in Figure 1. The Raman spectra were acquired at 12, 18 and 24 hpi, respectively. The results were analyzed and presented as mentioned in Figure 1.

**
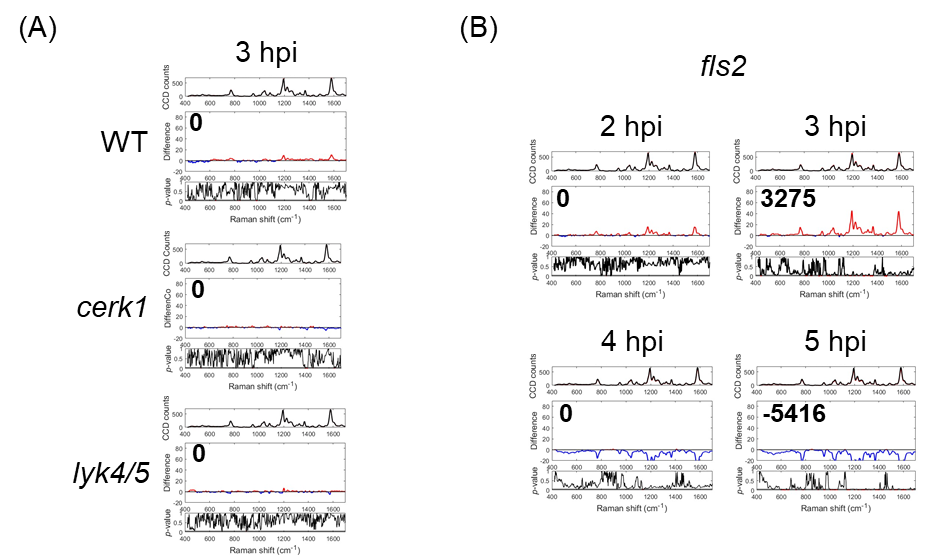
Supplementary Figure 4. Chitin-induced ERI before 6 hpi.** Arabidopsis plants were infiltrated with 5 µM chitin and analyzed by Raman spectroscopy a mentioned in Figure 1. The Raman spectra from WT, *cerk1*, and *lyk4/5* (A) were collected at 3 hpi; while that from *fls2* (B) were collected at 2, 3, 4, and 5 hpi and shown.


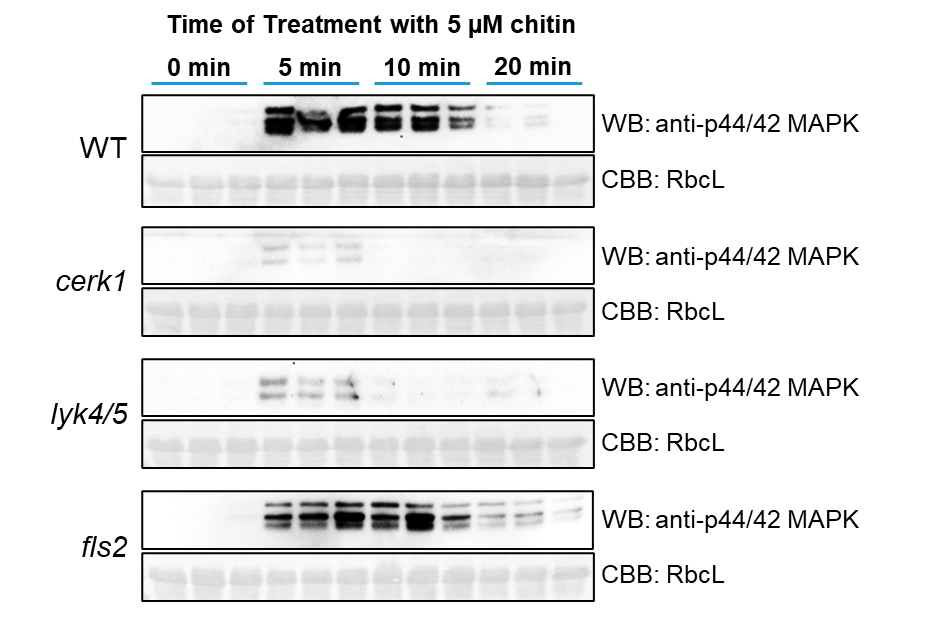


**Supplementary Figure 5. MAPK activation in WT and receptor mutants upon chitin treatment.** Wild type (WT, Col-0), *cerk1*, *lyk4/5* and *fls2* mutants were infiltrated with 5 µM chitin solution. Leaf samples from 3 independent plants were collected at 0, 5, 10 and 20 min after infiltration. Total protein extracts were analyzed by western blots using Phospho-p44/42 MAPK (Cell Signalling technology, Cat#4370) antibody. The large subunit of Rubisco (RbcL, 55 kDa) stained by Commassie Brillant Blue (CBB) served as the loading control.


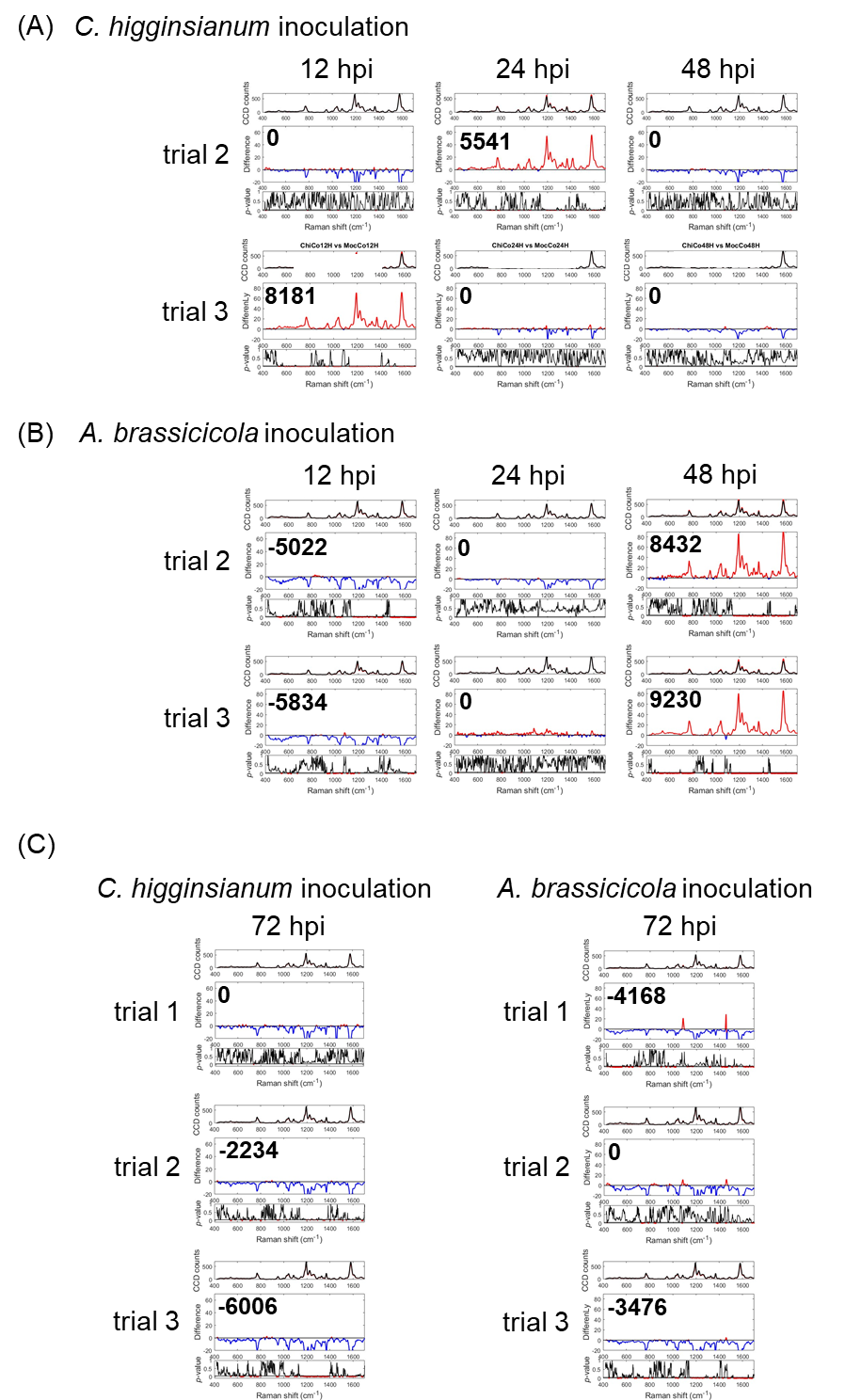


**Supplementary Figure 6. Raman spectroscopic analysis of *A. thaliana* inoculated with fungi*.*** Results from the two additional replicated trials presented in Figure 2 are shown. Three-week-old WT Arabidopsis plants were inoculated with (A) *C. higginsianum* (5 × 10^6^ spores/mL) or (B) *A. brassicicola* (10^7^ spores/mL) and leaf samples were collected at 12, 24, or 48 hpi. Each IRI represents data from 3 to 5 biological replicates. (C) Spectra from 3 independent trials obtained at 72 hpi for the indicated fungal inoculation are shown.


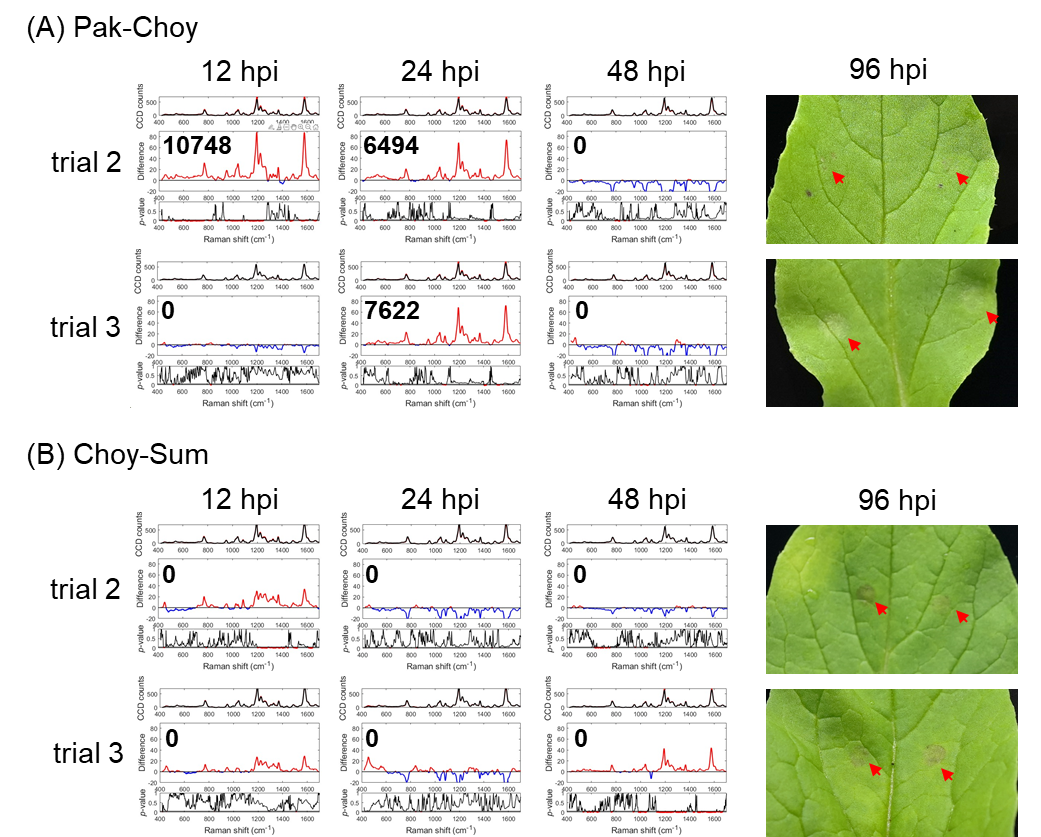


**Supplementary Figure 7. Raman spectroscopic analysis of two *Brassica* vegetables inoculated with *Colletotrichum higginsianum****.* Results from the 2 additional replicated trials presented in Figure 3A and 3B are shown. Twenty-five-day-old Pak-Choy (A) and Choy-Sum (B) were inoculated with *C. higginsianum* (5 × 10^6^ spores/ml) and samples were collected at 12, 24 or 48 hpi. Each IRI represents the results from 3 to 5 biological replicates.


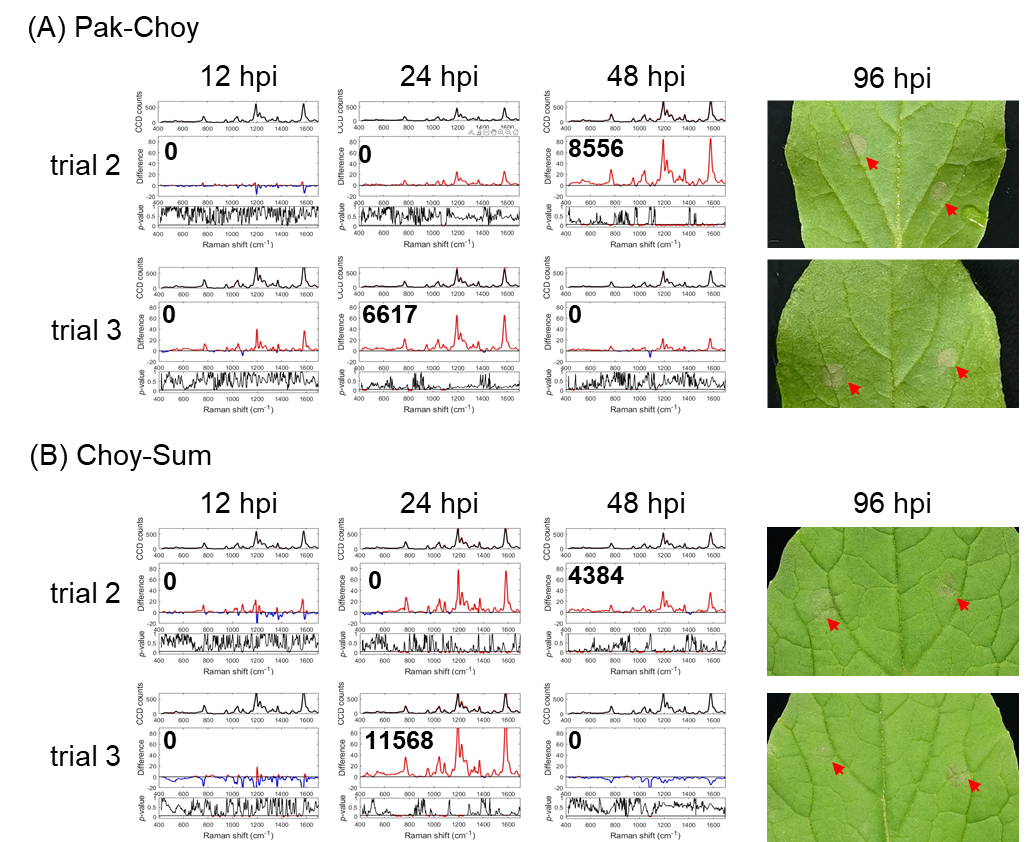


**Supplementary Figure 8. Raman spectroscopic analysis of two *Brassica* vegetables inoculated with *Alternaria brassicicola*.** Results from the two additional replicated trials presented in Figure 3C and 3D are shown. Twenty-five-day-old Pak-Choy (A) or Choy-Sum (B) were inoculated with *A. brassicicola* (10^7^ spores/ml) and leaf samples were collected at 12, 24 or 48 hpi. Each IRI represents the results from 3 biological replicates. Images on the right show disease symptoms at 96-120 hpi**.**

**
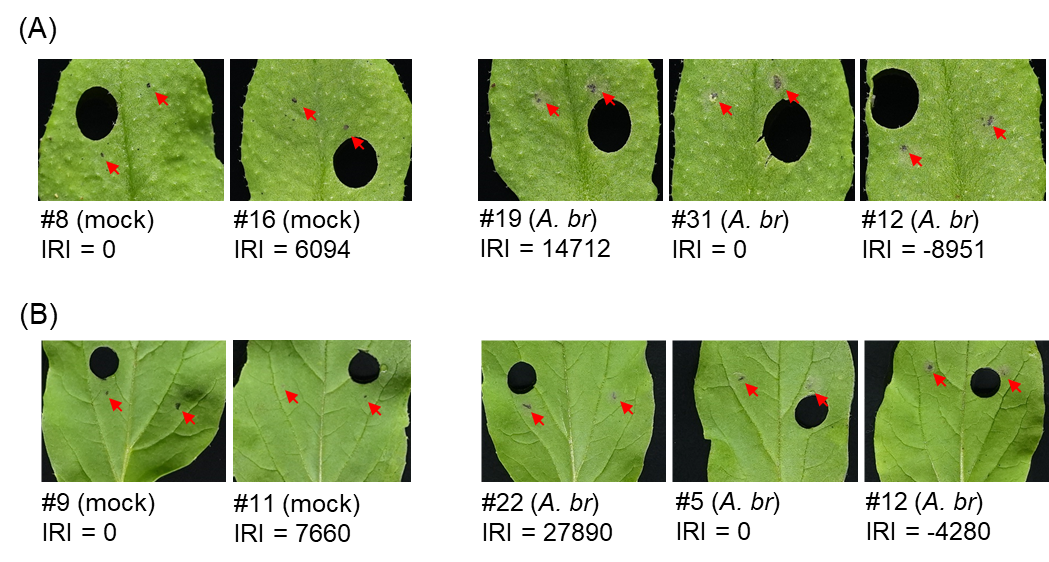
**

**Supplementary Figure 9. The symptom of fungal infection on Arabidopsis or Pak-Choy in a randomized controlled trial.** As mentioned in Figure 4, a total of 22 and 20 WT Arabidopsis plants (A), or a total of 21 and 19 Pak-Choy plants (B) were inoculated with *A. brassicicola* (A.br) or water (mock), respectively**.** The inoculated region was marked by a black dot and indicated by a red arrow; the Arabidopsis and Pak-Choy leaf discs were excised at 48 and 24 hpi, respectively, for analysis by Raman spectroscopy.


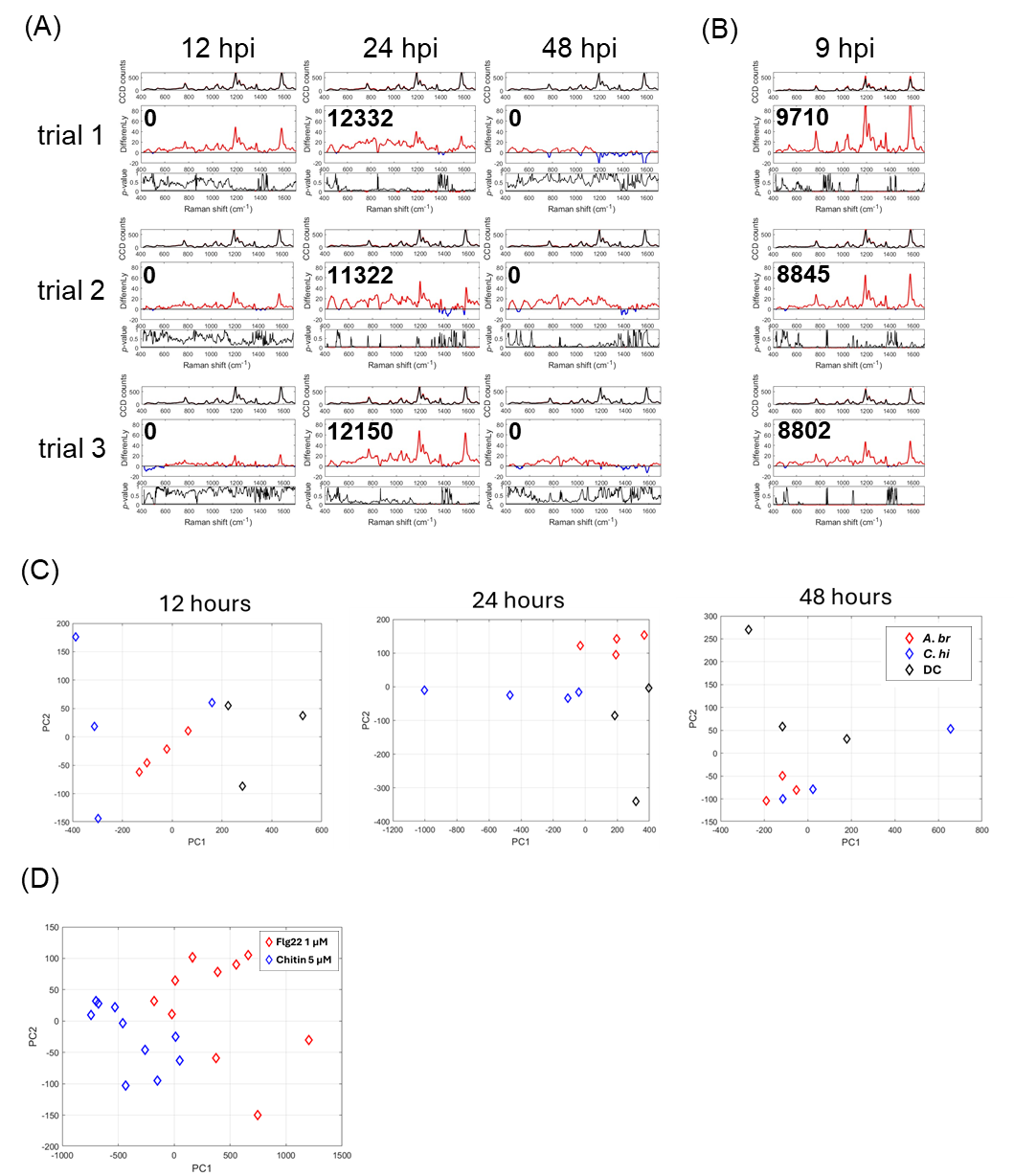


**Supplementary Figure 10. Raman spectroscopic analysis of WT Arabidopsis inoculated with *Pseudomonas syringae pv.* tomato DC3000 or Flg22.** WT Arabidopsis was inoculated with (A) *P. syringae* DC3000 (5 × 10^5^ cfu/mL) or treated with 1 µM Flg22 (B). Leaf samples were then collected at indicated time point and analyzed by Raman spectroscopy. Each ERI or IRI represents the results from 3 biological replicates**.** (C) Principal component analysis (PCA) method to distinguish the fungal infection and bacterial infection at 12, 24 and 48 hours. (D) Principal component analysis (PCA) method to distinguish the treatment of Flg22 and chitin. *C.hi*, *C. higginsianum* inoculated leaves; *A.br*, *A. brassicicola* inoculated leaves. DC, *Pseudomonas syringae* pv. tomato DC3000 inoculated leaves; Flg22 1µM, 1 µM Flg22 treated leaves; Chitin 5 µM, 5 µM Chitin treated leaves.


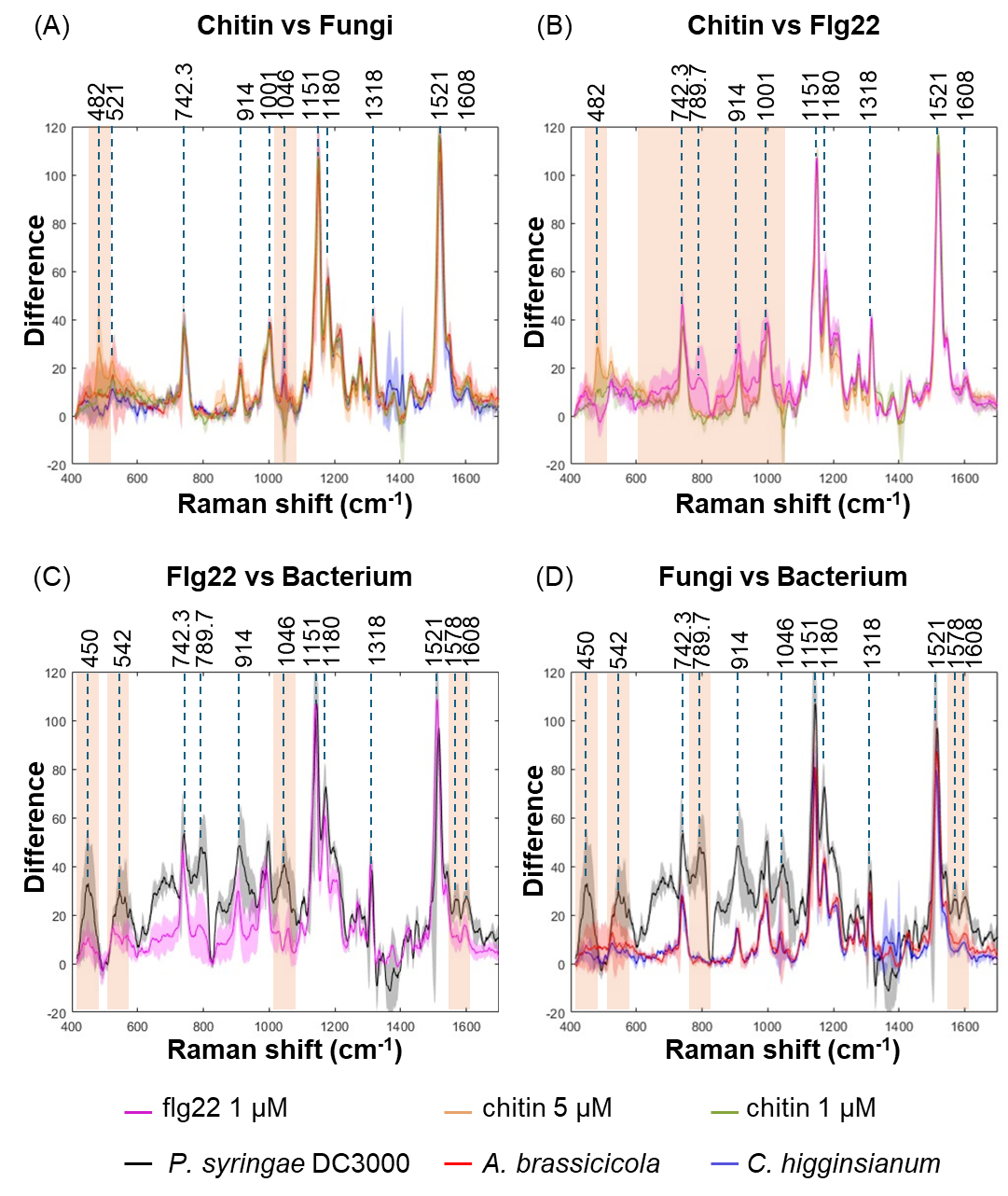


**Supplementary Figure 11. Raman spectral differences and internal variation between bacterial and fungal infection.** The spectral overlapping was performed as mentioned in Figure 5. The spectral overlapping from chitin treatment and fungal infection (A), chitin and Flg22 treatment (B), Flg22 treatment and Bacterial infection (C), as well as Fungal and bacterial infection (D) were presented. The Shadow along with each spectrum indicated the variation in triplicated experiments. Orange square shadow indicated the featured band in respective comparison.


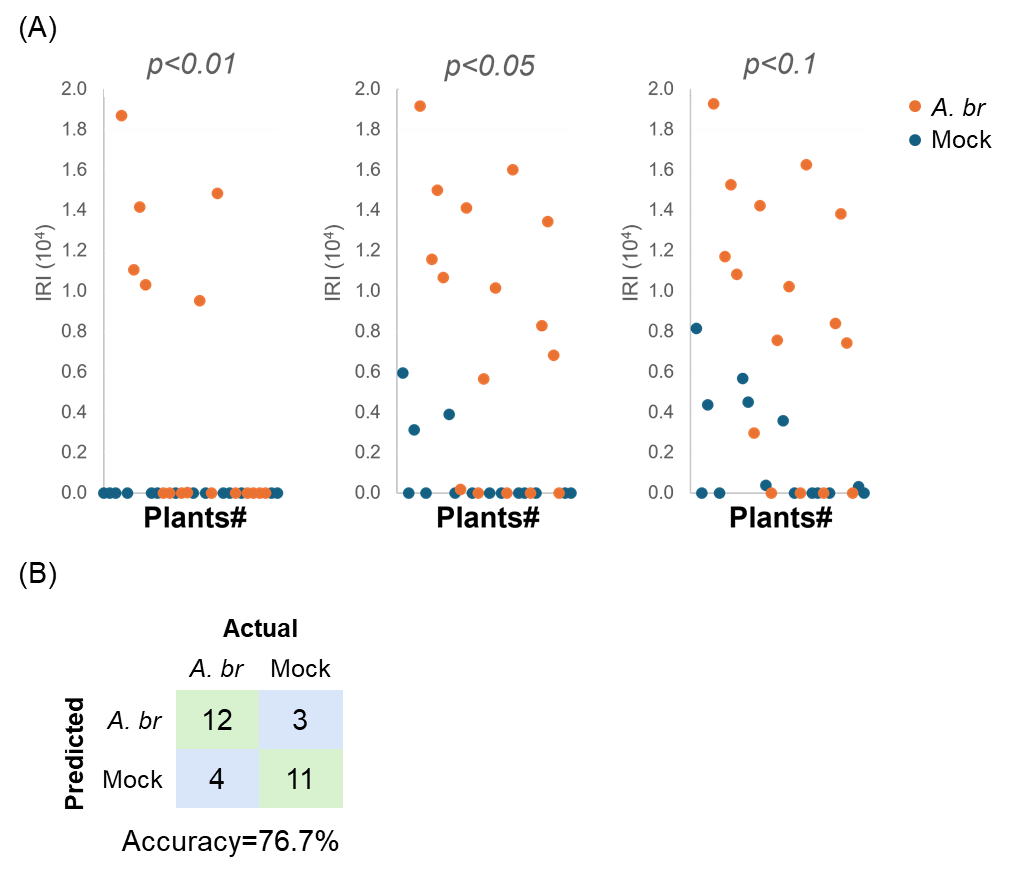


**Supplementary Figure 12. The second randomized control trial verify the application of Raman spectroscopy to detect potential fungal infection on Arabidopsis.** A replicated trial of experiments in Figure 4 are shown. A total of 16 and 14 WT Arabidopsis plants were subjected to *A. brassicicola*-inoculation (*A.br*) and mock-treatment (mock), respectively. The mean spectrum from each plant was compared to that from an independent trial comprising three standard mock-treated plants. The IRI of each plant was calculated at a significance level of *p*<0.01, *p*<0.05 and *p*<0.1 in t-test. (B) Confusion matrix showing the performance of the diagnostic method.
